# A Novel Online Data-Driven Algorithm for Detecting UAV Navigation Sensor Faults

**DOI:** 10.3390/s17102243

**Published:** 2017-09-29

**Authors:** Rui Sun, Qi Cheng, Guanyu Wang, Washington Yotto Ochieng

**Affiliations:** 1 College of Civil Aviation, Nanjing University of Aeronautics and Astronautics, Nanjing 211106, China; qi_cheng@outlook.com (Q.C.); guanyu_wang@outlook.com (G.W.); w.ochieng@imperial.ac.uk (W.Y.O.); 2 Centre for Transport Studies, Imperial College London, London SW7 2AZ, UK

**Keywords:** online, data-driven, navigation sensor fault detection, adaptive neuron fuzzy inference system

## Abstract

The use of Unmanned Aerial Vehicles (UAVs) has increased significantly in recent years. On-board integrated navigation sensors are a key component of UAVs’ flight control systems and are essential for flight safety. In order to ensure flight safety, timely and effective navigation sensor fault detection capability is required. In this paper, a novel data-driven Adaptive Neuron Fuzzy Inference System (ANFIS)-based approach is presented for the detection of on-board navigation sensor faults in UAVs. Contrary to the classic UAV sensor fault detection algorithms, based on predefined or modelled faults, the proposed algorithm combines an online data training mechanism with the ANFIS-based decision system. The main advantages of this algorithm are that it allows real-time model-free residual analysis from Kalman Filter (KF) estimates and the ANFIS to build a reliable fault detection system. In addition, it allows fast and accurate detection of faults, which makes it suitable for real-time applications. Experimental results have demonstrated the effectiveness of the proposed fault detection method in terms of accuracy and misdetection rate.

## 1. Introduction

Unmanned Aerial Vehicle (UAV) applications have been increasing in recent years, including surveillance, reconnaissance, search/destroy missions, aerial photography, and disaster monitoring [1]. Nevertheless, with the rapid development of these applications, some of which are safety critical, UAV flight safety has become a critical issue. UAV navigation sensors are a key component of its flight control system, necessitating that mechanisms are in place to ensure that faults are detected in a manner that ensures safety. However, because UAVs operate in changing and complex environments, it can be difficult to predict all possible faults. In addition, the highly dynamic operational environment requires faults to be detected in real-time. Therefore, due to the high importance of detecting navigation sensor faults there is a need to design a reliable algorithm that can extract the failures from the real-time UAV states fast, accurately, and with low misdetection and low false alarm rates.

In general, UAV navigation sensors faults can be classified into three types: point, contextual, and collective [2]. A point fault occurs when a data instance is shown as invalid; for example, a fault could cause the Global Positioning System (GPS) output to display a latitude value that is out of the range of the valid UAV latitude value. A contextual fault means that although a particular data instance may be valid on its own, it is considered as invalid with respect to a certain context. For example, the GPS receiver may output a velocity value of 0, which would be valid for the UAV on the ground but not in flight. A collective fault means that values are considered as valid individually, but not when put together collectively. For example, if the altimeter is stuck when a UAV’s altitude is increasing, the altimeter outputs values that while individually valid (i.e., each value is within the range of valid output values and valid in the context of the UAV being airborne) are not collectively valid since they are unchanged while other sensors report the UAV to be climbing. The fault detection algorithm presented in this paper is designed to detect these three types of navigation sensor faults as they manifest in the position domain through excessive position error. 

Specifically, there are a variety of ways in which the position error from an integrated navigation system can be excessive. These include system/sensor faults and those induced by the operational environment. Reference is made to Global Navigation Satellite System (GNSS) faults, including those due to malfunction in the satellite clock, incorrect modelling of the orbits, ionization of satellite payload silicon material, and inter-channel bias [3]. For the Inertial Measurement Unit (IMU) system, there are also several types of failure modes, including hardware and software faults and those specific to Micro-Electromechanical System (MEMS) technology failures (e.g., excessive scale factor, drift, and ageing). Furthermore, the sensor integration process can result in faults. These faults have the potential to result in excessive position error being generated by the Kalman Filter (KF) used for integration. The approach in this paper is designed to monitor the output of the KF (i.e., the position error) and thereby account for any faults that may be present in the data. Such faults include not only sensor specific ones but also those related to the operational environment (e.g., UAV operation in GNSS denied environment in which an outage or reduction of satellites or poor geometry contribute to degraded performance by the integrated positioning system).

In general, fault detection algorithms can be classified into three types: model-based, knowledge-based, and data-driven-based approaches [4]. For the classic model-based fault detection and diagnosis, the research could be classified into two distinct and parallel communities [5], i.e., the Fault Detection and Isolation (FDI) community and Diagnosis (DX) community. FDI is based on control theory and statistical decision making [6,7,8], while the DX is based on soft computing and artificial intelligence [9]. The concepts and assumptions for the two communities are quite different. Details on these techniques and approaches can be found in [5]. For the mode- based approach, Cork and Walker [10] developed an Interactive Multiple Model and Unscented Kalman Filter (IMM-UKF) estimation based UAV anomaly detection algorithm, which is focused especially on inertial sensor faults and their effect on the estimation of the control and performance of the UAV system. The initial results demonstrated the effectiveness of the designed algorithm in the UAV positioning error detection in specific scenarios. However, the algorithm has failure mode-mismatch errors, when applying multiple models. 

The knowledge-based approaches are based on the predefined rules (if-then) sentences. Bu et al. [11] introduced a UAV navigation sensor fault detection-based particle filter and fuzzy logic model. However, its performance is limited in terms of the false alarm rate and processing speed due to the very high computational load of the particle filter algorithm. Furthermore, the fixed rules in the fuzzy logic model have the impact of decreasing the performance of fault detection, as it is not able to detect the effect of unknown faults. 

The data-driven algorithms are based on the statistical information (usually obtained from the training results) to detect outliers and label them as the faults. For the data-driven approaches, Lin et al. [12] designed an algorithm to detect UAV sensor faults based on the monitoring of the internal and external sensor readings, including the navigation sensor data. The Mahalanobis distance was then calculated in order to identify the significant standard deviations within the collected data. This algorithm, however, could only be operated after fine tuning of the measurements to be within a certain threshold in the offline mode. Furthermore, the validity of the algorithm in the field has not been verified. Khalastchi et al. [4,13] proposed a data-driven approach for fast and accurate autonomous machine fault detection. The Mahalanobis distance was again used to evaluate sets of correlated attributes generated based on filter estimations from real-time in-flight navigation sensor parameters. It is claimed that the faults could be effectively detected based on a threshold determined directly from the distance residual size. A warning is triggered if the distance residual exceeds a specified threshold. A key limitation of this method is that it is difficult to adjust the threshold level to maintain a balance between accuracy and false alarm rate. 

From the literature above, although the classic model-based and knowledge-based algorithms are widely used in many applications, they have high model and rule dependencies, and, therefore, are unable to detect unknown or non-modelled faults. In addition, the classic fault detection algorithm performance will always be suboptimal for complex UAV navigation systems, due to the high computational effort required. Data-driven algorithms, meanwhile, exhibit superior ability in terms of fast response and flexibility based on statistical information.

Accordingly, this paper presents a novel data-driven Kalman Filter (KF)—Adaptive Neuro Fuzzy Inference System (ANFIS)-based algorithm for the detection of UAV navigation sensor faults. This method integrates the online data-driven detection cycle with the KF residuals and an ANFIS-based fault detector. The advantages over the state-of-the-art are: (1) Model free—without the need to consider the nature of the faults or models for the complex UAV navigation system, as required for the model-based approach in [10]; (2) Adaptive rules extraction—by employing a frequently updated training database online, unlike the traditional rule-based approach [11]. The latest features of all types of faults (point, contextual, and collective) present in the database are used for the extraction of fault judgement rules; (3) Robust fault detection decision making and more detailed analysis of the algorithm—the proposed KF residual with the ANFIS-based decision-making method is more robust compared to the simple Mahalanobis distance-based detector in [4,12,13], and faster than the Particle Filter (PF)- and Fuzzy Inference System (FIS)-based algorithm in [11]. In addition, field test results are presented and the relationship between the size of the sliding window and the fault detection performance is investigated in the paper. 

The contributions of the paper are as follows:

(1) Development of a novel data-driven KF residual analysis with ANFIS-based fault detection algorithm for fast online detection and low misdetection rate.
(2) The algorithm’s training database is updated online to ensure that it always contains the latest features of point, contextual, and collective faults.
(3) Novel adaptive online training strategies are employed for the extraction of ANFIS rules to ensure timely and accurate fault detection.

The rest of the paper is organized as follows. The online data-driven ANFIS-based algorithm for the detection of UAV navigation sensor faults is presented in Section 2. Simulation and field experiments to evaluate the proposed algorithm are presented in Section 3 and Section 4, respectively. Finally, the paper is concluded in Section 5 by a summary of the main findings.

## 2. Online Data-Driven ANFIS-Based Algorithm 

The system overview is presented in Section 2.1. The three parts of the fault detection model are: (i) Database creation, described in Section 2.2; (ii) ANFIS-based fault decision model, described in Section 2.3; and (iii) Online data-driven fault detection cycle, described in Section 2.4.

### 2.1. System Overview

**Figure 1:**
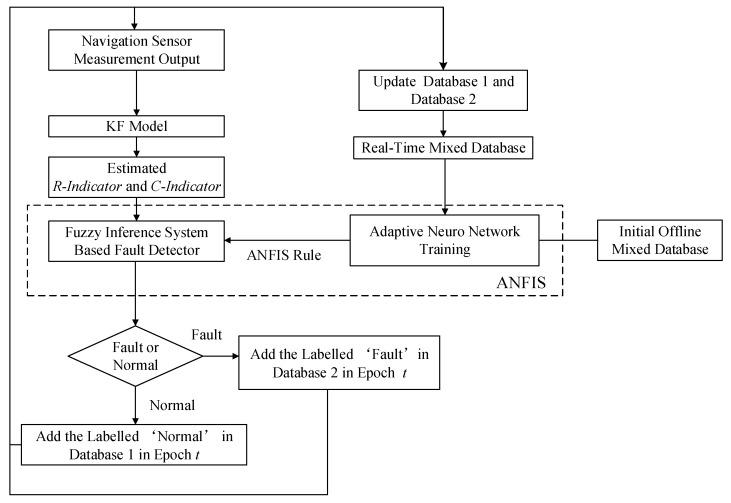
Overview of the algorithm. ANFIS, Adaptive Neuron Fuzzy Inference System; KF, Kalman Filter.

The flowchart for the online data-driven UAV navigation sensor fault detection is presented in Figure 1. 

Firstly, the initial offline mixed database is created with both “normal” and “fault” data collected and labelled from the UAV navigation sensors. The “normal” data are stored in Database 1 and the “fault” data in Database 2. Database 1 and Database 2 are then combined to form a mixed database. The data in the mixed database are used for ANFIS rule training and updated online by means of the fault detection cycle (described in Section 2.2). Afterwards, the real-time online navigation sensor measurement states are used as the input to the KF model to output real-time state estimations. Based on the KF estimations, the KF residual, $\Delta r_{t}$ is then calculated for fault detection. The *R-Indicator* and *C-Indicator* are further developed based on the $\Delta r_{t}$ to feed the ANFIS system for the designed fault indicator output, noted as the *F-Indicator*. In particular, the *R-Indicator* is developed based on the sliding window of the $\Delta r_{t}$ for three-axes states of the navigation sensor and the *C-Indicator* is calculated based on the Pearson’s correlation test and sliding window of the $\Delta r_{t}$ for three-axes states of the related navigation sensor [11]. The fuzzy rules and membership functions for the ANFIS are updated in real-time based on the adaptive neuro network training. Finally, based on the value of the *F-Indicator*, the status is determined to be either “normal” or “fault”. Accordingly, the newly determined status of the data is used to update the database for the next iteration (described in Section 2.3 and Section 2.4).

### 2.2. Database Creation

The previously experienced data for the navigation sensors are collected, cleaned, and labelled to create the initial offline database. The initial databases are represented in Equations (1)–(3).

(1) $$D1_{0}=\left\{N_{0-q+1},N_{0-q+2\;}\ldots ,N_{0}\right\}$$

(2) $$D2_{0}=\left\{F_{0-q+1},F_{0-q+2}\;\ldots ,F_{0}\right\}$$

(3) $$D_{0}=\left\{D1_{0},D2_{0}\right\}$$

The initial Database 1 is expressed as $D1_{0}$ in Equation (1), where, *q* is the length of the database. The data noted as N in *D*1 are always *nominal*. Accordingly, the initial fault data, including different types of faults, are cleaned and labelled and stored in Database 2, see Equation (2), where, *q* is the length of the database. The data noted as F in *D*2 are always classified as *fault*. The initial mixed database for the algorithm process is therefore expressed as $D_{0}$ in Equation (3). 

In the general case, the databases in any time epoch *t* could be expressed as follows.

(4) $$D1_{t}=\left\{N_{t-q+1}\;,N_{t-q+2}\;\ldots ,N_{t}\right\}$$

(5) $$D2_{t}=\{F_{t-q+1},F_{t-q+2}\;\ldots ,F_{t}\}$$

(6) $$D_{t}=\left\{D1_{t},D2_{t}\right\}$$

where, *t* ≥ 0 and *q* ≥ 1. 

### 2.3. ANFIS-Based Fault Detection Model

**Figure 2:**
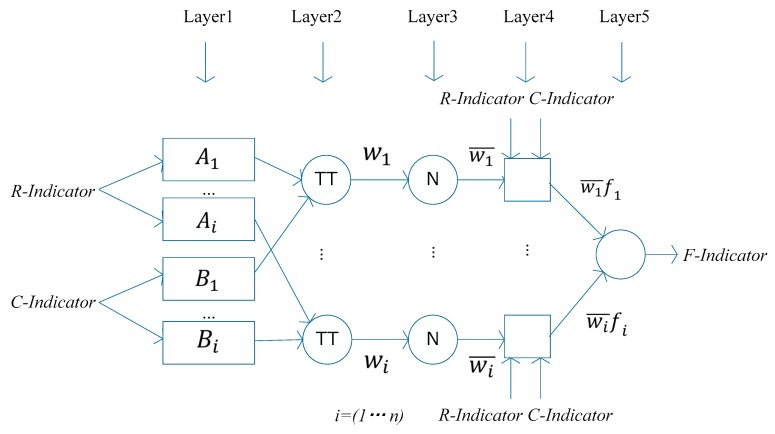
The structure of the designed ANFIS-based fault detection system.

ANFIS is an integrated algorithm by combining Neural Network (NN) architectures with Fuzzy Inference Systems (FISs). By employing the advantages of both FISs (i.e., their transparency and use of expert knowledge in their structure) and NNs (i.e., their fast learning capability), it is possible to extract the fuzzy rules and establish the adaptive membership function from the experienced input data based on neuro network training [14]. Figure 2 depicts the structure of the designed ANFIS-based fault detection system, which implements a first order Sugeno fuzzy model. 

The *R-Indicator* and *C-Indicator* are the two inputs into the ANFIS, and the *F-Indicator* is the output of the system. In total, the system is designed in five layers. A typical if-then rule in ANFIS can be expressed as:

If *R-Indicator* is A1 and *C-Indicator* is B1, then

(7) $$f_{1}=p_{1}\times A1+q_{1}\times B1+r_{1}$$

where the parameters defining the membership functions $A_{1}$, $B_{1}$, along with $p_{1}$, $q_{1},$ and $r_{1},$ are modified during the training. The description of each layer in ANFIS is as follows:

**Layer 1.** Assumes every node i in this layer is a square node with a node function. $O_{i}^{1}$ is the membership function of $A_{i}$. In our case, the initial membership functions of the input variables are set as the Gaussian based on the characteristic of the input information, as follows:

(8) $$O_{i}^{1}=\mu A_{i}\left(x\right)=Gaussian\;\left(Indicator;\sigma_{i},c_{i}\right)=e^{-\frac{{\left(Indicator-c_{i}\right)}^{2}}{2\sigma_{i}^{2}}}$$

where, Indicator represents the value of *R-Indicator* or *C-Indicator*, $c_{i}$ is the parameter to determine the centre of the membership function, and $\sigma_{i}$ determines the width of the curve. The parameters defined in this layer are the premise parameters.

**Layer 2.** Each node in this layer calculates the firing strength of each rule via multiplication. And T-norm operators are used here, given by,

(9) $$O_{i}^{2}=w_{i}=\mu A_{i}\left(x\right)\times \mu B_{i}\left(y\right),\;i=1,\;2,\;3$$

**Layer 3.** The ith node of this layer calculates the ratio of the ith rule’s firing strength to the sum of the firing strength of all rules. 

**Layer 4.** The multiplication of the input from Layers 3 and 1 is implemented. The parameters in this layer are therefore consequent parameters. 

**Layer 5.** The overall outputs as the summation of all incoming signals are computed. The grid partition method is employed for the initial FIS generation. The premise parameters in Layer 1 and the consequent parameters in Layer 4 are tuned until the desired response of the FIS is achieved during the learning process. Based on the training results of the ANFIS, the rule surface for the fault detection is established.

### 2.4. Online Data-Driven Fault Detection Cycle

The integration is performed through loose coupling, and therefore the monitored state is $x_{t}=\left\{GPSposition,\;acclerameter,\;gyro\ldots \;\right\}$, which are the inputs to the KF system. The KF then computes the estimated state ${\hat{x}}_{t}$. The KF is one of the most powerful data processing tools, and has been widely used in a range of the fault detection applications for years [15]. KF-based GNSS/INS integration and its application for fault detection is not new, and proof of convergence can be found in a number of sources including [16,17,18]. 

The KF residual, which reflects the discrepancy between the KF estimated state and the actual measured state, are generated as (10).

(10) $$r_{t}=y_{t}-{\hat{x}}_{t}$$

where, $r_{t}$ is the KF residual in time epoch t, $y_{t}$ is the measurement of UAV navigation sensor state at time epoch *t*, and ${\hat{x}}_{t}$ is the KF estimated state at time epoch *t*. The residual should be close to zero if the model is error free.

The difference in the residuals over time, $\Delta r_{t}$ is calculated to form a basis parameter for the fault detection in (11).

(11) $$\Delta r_{t}=r_{t}-r_{t-1}$$

where, $r_{t}$ is the KF residual in time epoch t and $\Delta r_{t}$ is the difference between the KF residual in time epoch t and t−1. The *R-Indicator* and *C-Indicator* are further developed based on the $\Delta r_{t}$ to feed the ANFIS system in order to generate the designed *F*-*Indicator* output.

The fault status output from the ANFIS is classified as either “Normal” or “Fault”. If the detected result $x_{t}$ is recognized as “Normal” in the time epoch *t*, the detected “Normal” data $x_{t}$ is named as $N_{t}^{'}$ and added to $D1_{t-1}$. At the same time, the end data $N_{t-q}$ in $D1_{t-1}$ is deleted. If the detected results are recognized as “Fault”, however, $x_{t}$ is named as $F_{t}^{'}$ and added to $D2_{t-1}$. The end data $F_{t-q}$ in the $D2_{t-1}$ are correspondingly deleted. The databases before the update are expressed as follows.

(12) $$D1_{t-1}=\left\{N_{t-q}\;,N_{t-q+1}\;\ldots ,N_{t}\right\}$$

(13) $$D2_{t-1}=\left\{F_{t-q},F_{t-q+1}\;\ldots ,F_{t}\right\}$$

(14) $$D_{t-1}=\left\{D1_{t-1},D2_{t-1}\right\}$$

where, $D1_{t-1},\;D2_{t-1},\;D_{t-1}$ are the “normal”, “fault”, and mixed databases in the time epoch *t* – 1 before update, respectively. 

As the detected status of $x_{t}$ would be either “normal” or “fault” in a specific epoch, the update of the databases has two scenarios or cases. If the detected results are recognized as “normal”, the updated databases in the time epoch *t* are expressed as follows:

(15) $$D1_{t}=\{N_{t-q+1},\;N_{t-q+2}\;\ldots ,N_{t}{}^{'}\}$$

(16) $$D2_{t}=D2_{t-1}$$

(17) $$D_{t}=\left\{D1_{t},D2_{t}\right\}$$

If the detected results are recognized as “fault”, the updated databases in the time epoch *t* are as follows:

(18) $$D1_{t}=D1_{t-1}$$

(19) $$D2_{t}=\left\{F_{t-q+1},\;F_{t-q+2}\;\ldots ,F_{t}{}^{'}\right\}$$

(20) $$D_{t}=\left\{D1_{t},D2_{t}\right\}$$

where, $D1_{t},\;D2_{t},\;D_{t}$ are the “normal”, “fault”, and mixed databases in the time epoch *t* after update, respectively.

The created real-time mixed database $D_{t}$ is used for continued ANFIS training.

The up-to-date rules extracted from the training are used for the fault detection. Considering the requirement of fast detection and a low false alarm rate, the training for the ANFIS is designed to be adaptive, which means that the training is not carried out for every iteration cycle. Instead, the ANFIS training is carried out only when a certain amount of “fault” labelled data is accumulated in Database 2. This means that the neuro network-based training is adaptively linked to the database update conditions, ensuring that the fuzzy logic rules are updated properly, while still allowing for fast detection. A detailed analysis is presented in the simulation section.

## 3. Simulation

**Figure 3:**
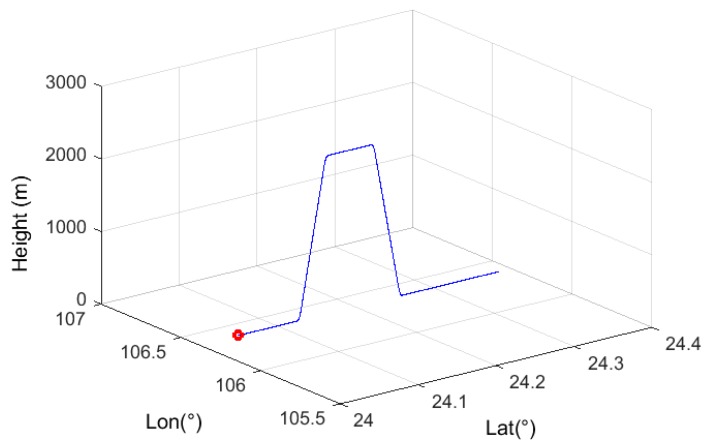
Simulated flight trajectory.

A simulation was carried out to test the performance of the designed algorithm in different scenarios. A 250 s UAV flight profile, including climbing, level flight, and descent, was generated with MATLAB (see Figure 3). The reference trajectory for the predefined route and the simulated GPS/IMU integration including periods of GPS outage and satellite loss of lock (representing environment induced faults) were generated. The three defined fault scenarios were: (i) a GPS outage in the ascent phase (30 s–40 s), (ii) a GPS outage in the level flight phase (100 s–110 s), and (iii) a GPS outage in the descent phase (220 s–230 s). Note that the algorithm developed monitors the impacts of these “faults” on the positioning error from the output of the KF-integrated GNSS/IMU system.

During the simulation, the Training Condition (TC) indicator was optimised for the online data-driven ANFIS fault detection algorithm. The TC is the indicator defined to represent the accumulated number of “fault” data for training. For example, if TC = 20, when the number of detected “fault” data in the *D*2 accumulates to 20, new training is initiated for the generation of rules to be used for the next iteration. 

**Figure 4:**
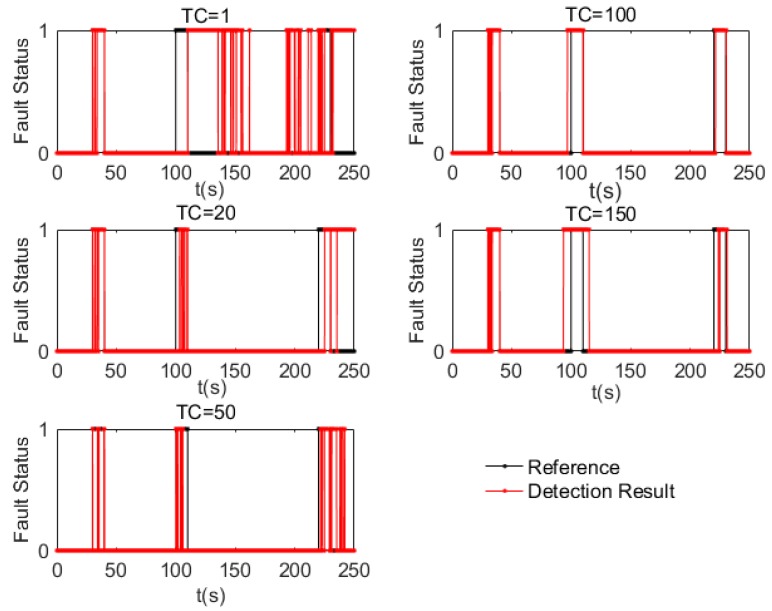
Fault detection results based on different Training Condition (TC) values.

Figure 4 shows the fault detection results with different TC values. Comparing the detection results with the reference, the best performance happens when TC = 100 and the worst when TC = 1. A confusion matrix of the fault and normal (1 and 0, respectively) detection results using the proposed online data-driven ANFIS-based algorithm with different TC values is presented in Table 1. Detection accuracy is calculated as the ratio (in percentage) of the number of correct detected activities to the number of total known activities, false alarm rate as the ratio of the number of false positive activities (0 but detected as 1) to the total number of detected faults and misdetection rate as the ratio of the number of false negative activities (1 but detected as 0) to the total number of actual faults. 

By considering the accuracy, misdetection rate, false alarm rate, and computation time, the best performance in terms of accuracy (97.7%), false alarm (13%), and time consumption (11 s) were all achieved at TC = 100. In addition, while the accuracy steadily increased and the false alarm rate decreased as the TC value was increased from 1–100, increasing the value of TC to 150 results in the performance deteriorating slightly compared to the performance at TC = 100. The reason for this is that when TC = 150, the rules are no longer current for accurate fault detection. The reason for the lowest performance at TC = 1 is that this high training frequency resulted in more inaccurate detection results being added to the updated database. The accuracy of the rules generated from the updated database is affected by the accumulated inaccuracy of the training data, resulting in accumulated errors in the whole fault detection cycle.

## 4. Initial Field Test

**Figure 5:**
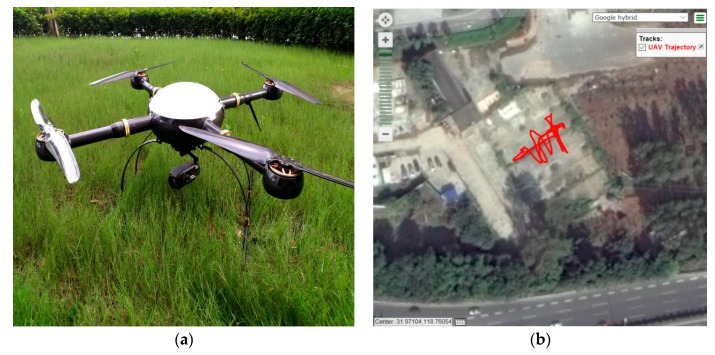
(**a**) The Unmanned Aerial Vehicle (UAV) used in the field test and (**b**) its test trajectory.

An initial field test was carried out to validate the effectiveness of the proposed data-driven ANFIS-based fault detection algorithm. The test site was close to Ningshuang Road, Nanjing City, China. The UAV used in the field test is from TopXGun Robotics and the flight route in shown in Figure 5. The UAV flight data was collected from around 10:12:00–10:19:00 Beijing time. The flight test was more complex than the simulated flight trajectory, as it not only included take-off and landing, but also roving in the air, in straight and curved manoeuvres.

The duration of the test was about 7 min and three operational environment-induced faults occurred due to GPS signal obstruction or observed satellite geometry changes. These three fault sessions are further defined in Table 2. The test data regarding the tri-axial states of the UAV was generated from GPS and IMU sensor measurements at a 10 Hz output rate, while the reference UAV manoeuvre data was generated by Real-Time Kinematic (RTK) GPS and IMU integration with forward and backward post-processing.

**Figure 6:**
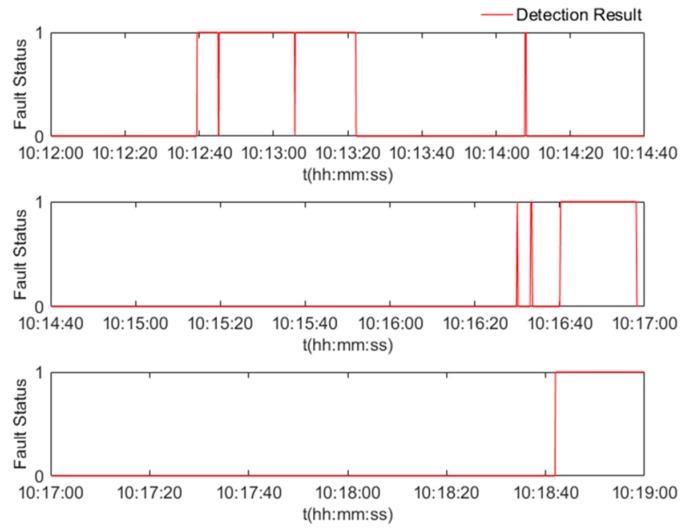
Fault detection results for the velocity fault in the field test.

An example of the velocity fault status with 10 Hz output rate is shown in Figure 6. It can be seen that the proposed algorithm detected the faults in the designed sessions. Table 3 further illustrates the related parameters for the performance of the algorithm and compares the proposed online data-driven ANFIS algorithm with the FIS-based UAV navigation sensor fault detection algorithm in [11]. From the detection results with real data, it is validated that the proposed data-driven ANFIS-based algorithm is superior to the FIS-based algorithm in [11], in terms of accuracy, false alarm rate, and misdetection rate. Specifically, with the proposed algorithm, the average misdetection rate was significantly reduced from 24.9% to 9%, the false alarm rate was reduced from 25.4% to 14%, and the accuracy improved from 82% to 92.6%. It is important to note that the proposed approach in this paper is data-driven (i.e., detection is based on the quality of the data), and therefore accounts for all sources of faults including those related to trajectory and environmental contexts.

The risk of hazardously misleading information (i.e., integrity risk) is the product of the probability of sensor/system failure and the probability of misdetection.

(21) $$P_{Integrity\;Risk}=P_{sensor/\;system\;Failure}\times P_{Misdetection}$$

Our research has shown that for the UAV delivery application, the required Integrity Risk (IR) is of the order of $\;10^{-5}$ per hour. If the sensor failure probability is assumed to be $10^{-4}$ per hour, as is the case for GPS satellites [19], then then probability of missed detection is 0.1. From our field test results, the misdetection rate is 0.08, which meets this requirement. However, the false alarm rate is still a bit high, and further work is planned to fine tune the algorithm. 

## 5. Conclusions

This paper presents a novel online data-driven ANFIS-based algorithm for UAV on-board navigation sensor fault detection, by combining the conditional online data training with KF and an ANFIS-based decision system. Compared to the traditional fault detection methods which have a high dependency on models and rules—and therefore are not effective at detecting unknown or non-modelled faults—the proposed online data-driven algorithm provides robust and flexible fault detection without the need for a priori models and rules. The proposed algorithm is the first based on a KF algorithm to obtain high accuracy residual estimations. Afterwards, the *R-Indicator* and *C-Indicator*, which capture the characteristics of the data (e.g., spatial and temporal), were developed to feed the ANFIS-based decision system. In addition, the proposed dynamic database creation algorithm ensures reliable fault detection in the presence of sudden changes in fault types. 

The field test results show that the developed fault detection model can provide improved detection results, with accuracy, false alarm rate, and misdetection rates of 92.6%, 14%, and 8%, respectively. This is better than the PF-FIS-based algorithm in [11] (which featured results of 82%, 25.4%, and 24.9%, respectively). From the application requirements, the misdetection rate achieved is acceptable for some UAV applications such as delivery. Research is underway to capture more representative data in order to further improve the performance of the data-driven algorithm, and, in particular, increase the accuracy and reduce the number of false alarms.

## Figures and Tables

**Table 1:** Confusion matrix of fault and normal (1 and 0, respectively) detection results based on the proposed data-driven algorithm with different training strategies.

		**Number of “Fault” Data Accumulated**									
Training Condition (TC)		TC = 1		TC = 20		TC = 50		TC = 100		TC = 150	
		**Algorithm Calculated Results**									
		1	0	1	0	1	0	1	0	1	0
Label Results	1	84	216	134	166	110	190	286	14	237	63
	0	721	1479	200	2000	70	2130	43	2157	117	2083
Accuracy		0.625		0.854		0.896		0.977		0.928	
False Alarm Rate		0.896		0.599		0.389		0.130		0.331	
Misdetection Rate		0.720		0.553		0.633		0.047		0.210	
Calculation Time Used		102s		13s		15s		11s		15s	

**Table 2:** Defined fault sessions and the time duration of the faults.

Session ID	Faults Start Time	Faults Stop Time
Session 1	10:12:39.5	10:13:20.2
Session 2	10:16:39.3	10:16:58.5
Session 3	10:18:41.1	10:19:00.0

**Table 3:** Fault detection performance in the defined sessions.

Session ID	Parameters	Proposed Algorithm (TC = 100) (%)	Algorithm from [11] (%)
Session 1	Accuracy	93.6	83.1
	False Alarm Rate	14.1	23.2
	Misdetection Rate	7.6	26.1
Session 2	Accuracy	91.3	82.5
	False Alarm Rate	15.4	25.8
	Misdetection Rate	8.3	20.4
Session 3	Accuracy	92.8	80.4
	False Alarm Rate	12.5	27.3
	Misdetection Rate	8.1	28.3
Average Performance	Accuracy	92.6	82
	False Alarm Rate	14	25.4
	Misdetection Rate	8	24.9
